# The Impact of Changes in Nutritional Policy on the Determinants of Child Stunting: Evidence From the 2010 and 2022 Living Conditions Monitoring Surveys in Zambia

**DOI:** 10.1002/fsn3.71794

**Published:** 2026-04-24

**Authors:** Richard Bwalya, Thomson Kalinda

**Affiliations:** ^1^ Department of Agricultural Economics and Extension University of Zambia Lusaka Zambia

**Keywords:** determinants, nutrition, Oaxaca decomposition, policy, rural, stunting, urban, Zambia

## Abstract

This study extends earlier work on the determinants of child stunting in Zambia by incorporating 2022 Living Conditions Monitoring Survey data, enabling assessment of nutritional change over a longer post‐policy period. It supplements the earlier paper by using 2022 LCMS data as opposed to 2015, thereby giving a longer period between the policy change and evaluation. We analyze changes in child height‐for‐age *z*‐scores (HAZ) in Zambia between 2010 and 2022, aligned with the 2011–2015 and 2017–2021 National Food and Nutrition Strategic Plans. National HAZ improved (Δ ≈ −0.92 SD), with gains in improved water (+26 pp), sanitation (+18.7 pp), dietary diversity (+0.78), and maternal schooling (+0.79); however, crowding (+0.08), the child‐to‐adult ratio (+0.13), and the food budget share (+9 pp) rose. Blinder—Oaxaca results indicate both endowments and returns contributed nationally. Rural areas achieved a smaller gain (Δ ≈ −0.60 SD), driven mainly by stronger returns—especially a steeper HDDS‐HAZ gradient—while endowments changed little. Urban‐dominant areas saw a larger gain (Δ ≈ −1.56 SD) due to both endowments (household structure, food spending) and returns, with the housing‐space coefficient partially offsetting progress. Overall, Zambia's multisectoral approach likely supported broad improvements, but sanitation gaps and urban environmental risks constrained impacts. Priorities: accelerate rural sanitation with hygiene SBC; protect diet quality via food‐system actions and social protection; and strengthen urban environmental health.

## Graphical abstract

Using LCMS data (2010, 2022) and Blinder–Oaxaca decomposition, this study shows that child height‐for‐age in Zambia improved (Δ ≈ −0.92 SD) due to both enhanced household conditions and stronger nutritional returns, with distinct rural and urban pathways, highlighting the need for improved sanitation, diet quality protection, and stronger urban environmental health interventions.

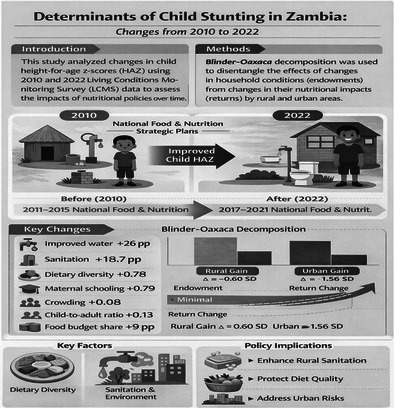

## 1 Introduction

**FIGURE 1:**
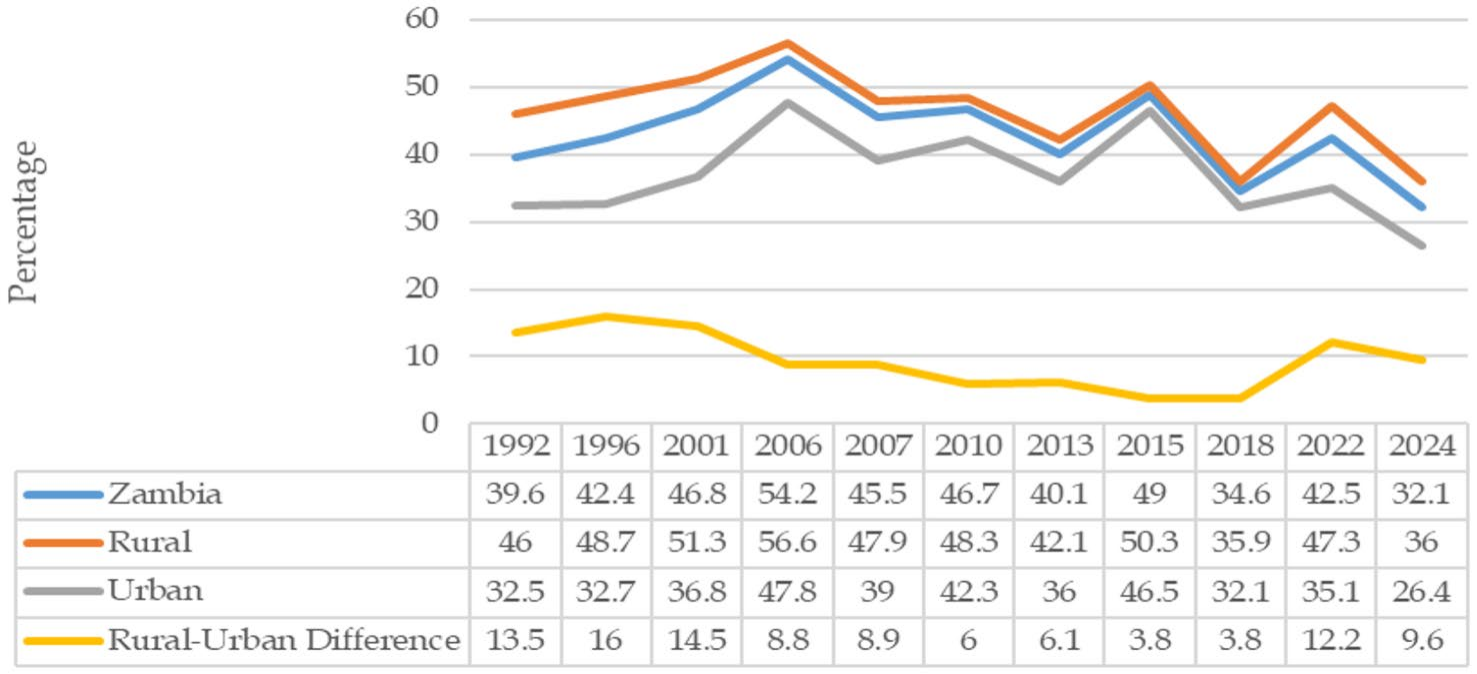
Rural–urban trends in child stunting, 1992–2024. *Sources:* University of Zambia et al. (1993); CSO et al. (1997); CSO et al. (2003); CSO et al. (2009); CSO et al. (2014); CSO, (2012); CSO (2016); CSO (2019); ZamStats (2023); ZamStats et al., (2024).

Ensuring adequate nutrition, especially among the low‐income groups, mothers, children, and vulnerable populations, has always been a serious challenge for Zambia. Although the country has shown some progress in reducing stunting over time (Figure 1), the rate remained high at 32.1% in 2024 (ZamStats et al. 2024) and way above the 22.8% average reported for the Southern African region (FAO et al. 2023). As with poverty, child malnutrition is more a rural than an urban phenomenon. The Zambia Demographic Health Survey (ZDHS) for 2024 shows that the proportion of rural children that are stunted is higher (36.0%) than that for urban children (26.4%). Malnutrition also poses a serious health challenge, as it is the cause of up to 52% of all deaths in under‐five children (MoH 2012). Estimates of the economic cost of malnutrition for Zambia are high, particularly for stunting, which does not only affect health (physical and cognitive development) but also negatively affects productivity in adulthood. For example, according to the Ministry of Health (MoH et al. 2017), if stunting levels remain unchanged for the period 2016 to 2027, future productivity losses related to stunting would be about US$18.315 billion.

Developing a viable investment and policy strategy for reducing child malnutrition in Zambia, as in most African countries, has remained elusive. The high levels of malnutrition have persisted despite various policies and programmes aimed at addressing malnutrition. Zambia's commitment towards improving nutrition dates back as far as 1967, when the National Food and Nutrition Commission (NFNC) Act was enacted by Parliament. This Act paved the way for the launch of the National Food and Nutrition Policy (NFNP) in 2008, which had its food and nutrition objectives and broad actions incorporated into the national development agenda through the Fifth National Development Plan (2005–2010), Sixth National Development Plan (2011–2015), Seventh National Development Plan (2017–2021) and Zambia's long‐term vision known as Vision 2030. The NFNP is implemented through five‐year National Food and Nutrition Strategic Plans (NFNSP). For this analysis, we focus on the NFNSPs developed in 2011 covering the period 2011–2015 as well as for 2017, covering the period 2017–2021. The NFNSP (2011–2015) was Zambia's first multi‐sectoral response to combat malnutrition and focused on 11 key operational strategic directions (OSDs) related to improving food and nutrition in the country, while the NFNSP (2017–2021) was Zambia's second multi‐sector five‐year strategic response to combating malnutrition. Although the NFNSP (2017–2021) has 9 OSDs that address discrete and overlapping problems which often require multisector solutions, it gives special priority to the first ODS, which is prevention of stunting in children under‐2 years of age (NFNC 2019).

The persistence of rural–urban disparities in child nutrition highlights the need for an enhanced understanding of the main drivers of rural–urban differences in nutrition outcomes. An important associated public health policy question is whether fundamentally different nutritional policies and interventions are required in rural and urban areas. Existing studies on child stunting in Zambia and similar low‐ and middle‐income countries have largely relied on cross‐sectional analyses to document associations between child nutrition outcomes and factors such as maternal education, household wealth, caregiving practices, and access to health and WASH services (Behrman and Wolfe 1984; Desai and Alva 1998; Fink et al. 2014). While this literature is informative, it provides limited insight into whether observed improvements in child nutrition reflect changes in households' underlying conditions or changes in the effectiveness with which these conditions translate into child growth. Moreover, relatively little empirical work has explicitly examined whether these pathways differ between rural and urban settings, despite well‐documented spatial disparities in stunting prevalence in Zambia (CSO et al. 2014; ZamStats et al. 2024).

This paper builds on an earlier paper that examines the impact of changes in nutritional policies on the underlying determinants of child stunting in Zambia by decomposing the observed gap in child stunting between urban and rural children (Bwalya and Kalinda 2023). The results show that differences in the levels of endowments between rural and urban areas accounted for most of the observed differences in stunting for the periods 2010 and 2015. This paper extends this analysis by decomposing the drivers of nutritional change in under‐five children between the periods 2010 and 2022, a period when Zambia adopted a multi‐sectoral approach to addressing child malnutrition (NFNC 2017). Using nationally representative Living Conditions Monitoring Survey data and a Blinder–Oaxaca decomposition framework (Blinder 1973; Oaxaca 1973; Jann 2008), the study distinguishes between changes attributable to shifts in key nutrition‐related determinants—such as dietary diversity, caregiving characteristics, and environmental health conditions—and changes in the strength of their associations with child growth. The analysis is conducted at the national level and separately for rural and urban areas to identify context‐specific drivers of nutritional change and to generate evidence relevant for the design of more targeted nutrition and health policies.

This study contributes new evidence by decomposing *changes* in child linear growth over a long policy‐relevant period (2010–2022), rather than focusing on static correlates of stunting. Unlike much of the existing literature, the analysis explicitly distinguishes between changes in underlying determinants and changes in their associations with child growth and compares these pathways across rural and urban settings.

## 2 Materials and Methods

### 2.1 Data Sources

The paper relies on Zambia Living Conditions Monitoring Survey (LCMS) data for 2010 and 2022, which is available from the Zambia Statistical Agency. Although similar studies have used Demographic Health Surveys (DHS), the LCMS is preferred as it enables researchers to examine the predictive power of a wide range of policy‐relevant explanatory variables, such as household food security, women's education and women's access to productive resources, children's access to health services and utilization, as well as water supply, sanitation and housing conditions, which are measured using nationally and sub‐national representative household surveys. The other motivation for using LCMS rather than DHS data is that although both data sets contain similar information on child malnutrition, the implementation of the LCMS surveys coincides more closely with the changes in nutrition policy in Zambia. In terms of sample design and coverage, the LCMS uses nationally representative cross‐sectional household surveys with varied sample sizes. While the 2010 survey was designed to cover a representative sample of about 19,300 non‐institutionalized private households residing in both rural and urban parts of the country, the 2022 survey was much smaller and designed to cover a representative sample of only 8520 households.

### 2.2 Conceptual Framework

**FIGURE 2:**
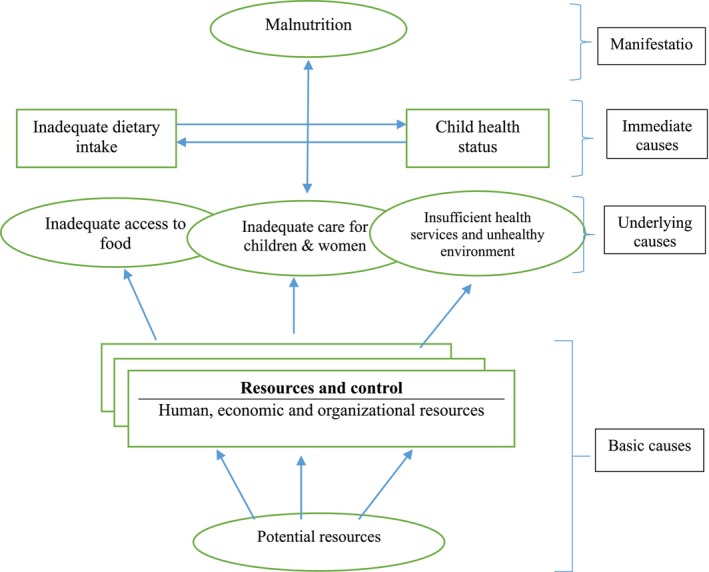
Conceptual framework guiding the empirical analysis. *Source:* UNICEF (1990).

The study uses the widely accepted framework developed by the United Nations Children's Fund (UNICEF) in the early 1990s (Figure 2) and has since been used in a number of studies on child malnutrition (UNICEF 1990). This conceptual framework, together with the variables available in our data sets, leads us to investigating the roles that the underlying determinants of child nutrition—household food security status, quality of care for mothers and children, and healthy environment and health services—have played in the nutritional status of both rural and urban children, as well as how these determinants have evolved in response to changes in food and national nutritional policies. It illustrates the hierarchical relationship between the immediate, underlying, and basic determinants of child nutrition status.

### 2.3 Measurement of Variables

The choice of explanatory variables was guided by the UNICEF conceptual framework for child nutrition (UNICEF 1990), existing empirical evidence on the determinants of child stunting (Black et al. 2013; Headey et al. 2017), and the availability of consistently measured indicators across the 2010 and 2022 Living Conditions Monitoring Surveys.

#### 2.3.1 Dependent Variable

Existing child nutrition studies have found different growth‐nutrition elasticities, depending on whether underweight prevalence (weight for age), wasting prevalence (weight for height), or stunting prevalence (height for age) is used as the dependent variable (Headey 2013). Linking back to the general objective of the study, which is to assess how the determinants of rural and urban malnutrition have evolved between the two periods in response to changes in the nutrition policy, height‐for‐age *z*‐scores (HAZ), also referred to as stunting, are used as an indicator of child nutritional status. Stunting is a good indicator of child nutrition and health as it reflects the effects of chronic nutritional deficiency.

#### 2.3.2 Measurement of Other Key Variables

##### 2.3.2.1 Household Food Security Variables

With regards to household food security‐related variables, we focus on those indicators that can be measured using Household Expenditure Surveys (HES) data. According to Smith and Subandoro (2007), each of these indicators addresses some aspect of the following definition of food security, adopted at the 1996 World Food Summit: “Food security … [is achieved] when all people, at all times, have physical and economic access to sufficient, safe and nutritious food to meet their dietary needs and food preferences for an active and healthy life” (FAO 1996). Specifically, two food security indicators that focus on diet quality and economic vulnerability are used. For diet quality, we use the household dietary diversity score (HDDS), which is a continuous variable defined as the number of food groups consumed from a list of 7 food categories as defined by the FAO (Kennedy et al. 2011). For economic vulnerability, which relates to people's ability to acquire food, we use the percentage of total household expenditure on food (food expenditure share).

##### 2.3.2.2 Care for Women and Children

The second category of underlying determinants, according to the conceptual framework, is care for women and children. This category includes mother‐specific and child‐specific variables. For the mother‐specific variables we use maternal education, which has long been associated with improved child nutrition outcomes (Alderman and Headey 2014; Behrman and Wolfe 1984; Desai and Alva 1998; Webb and Block 2004). We also use mother's age, which is not only associated with low birthweight and pre‐term birth (Du Plessis et al. 1997), but also with an increased risk of intrauterine restriction (Conde‐Agudelo et al. 2005). The variable “number of children under the age of five” is used as a proxy for birth spacing, which plays a key role in nutrition status among children under the age of five, with shorter birth intervals being associated with an increased risk of both stunting and underweight (Gribble et al. 2009). For child‐specific variables we include the child's age in months and the sex of the child.

##### 2.3.2.3 Healthy Environment and Health Services

The third category of underlying determinants is a healthy environment and health services. We use a number of indicators in this category, namely, access to improved water sources for drinking water, access to improved sanitation facilities, and crowding index. There is a significant body of literature that finds associations between access to piped water and reductions in the incidence of diarrhea as well as chronic undernutrition. For example, contaminated drinking water may jeopardize children's nutrition status as waterborne illnesses are the second most common causes of death for children under the age of five (UNICEF 2008). The World Health Organization estimates that 50% of malnutrition is associated with repeated diarrhea or intestinal worm infections from unsafe water or poor sanitation or hygiene (Ngure et al. 2014). Household density (measured by the crowding index) has long been viewed as both an indicator of low socioeconomic status and also as a stressful situation associated with high morbidity and mortality risks. Indeed, research shows that the crowding index (denoted by number of residents per room) is correlated with a wide range of pathological health outcomes (Freedman 1975; Baker et al. 1998). Household poverty status, defined as whether a household's consumption is below the poverty line or not, is also included as a major underlying determinant of child stunting, as it limits household access to adequate nutrition, health services, and improved sanitation—factors essential for optimal child growth and development (Black et al. 2013).

### 2.4 Analytical Approach

Motivation. Differences in child nutrition outcomes across groups (or over time) may arise because (i) groups differ in the levels of key determinants (covariate or endowment effects) and/or (ii) the same determinants are associated with outcomes with different strengths (coefficient or returns effects). To quantify each channel for changes in height‐for‐age *z*‐scores (HAZ) between 2010 and 2022, we apply a Blinder–Oaxaca decomposition (Blinder 1973; Oaxaca 1973).

#### 2.4.1 Reference Structure and Model Choice

In implementing the Blinder–Oaxaca decomposition, we report results using both the Black–White and Gray model specifications. The Black–White specification uses one group's coefficient structure as the reference, providing an interpretable benchmark for group‐specific comparisons, while the Gray specification employs pooled coefficients as a neutral reference that avoids privileging either group. Using both approaches addresses the index‐number problem inherent in decomposition analysis and allows assessment of the robustness of results to alternative reference structures. The consistency of findings across these specifications indicates that the main results are not driven by the choice of decomposition model.

#### 2.4.2 Conceptual Framework

We follow the household production framework (Becker 1965; Strauss and Thomas 1995), where households derive utility from consumption, leisure, and children's quality (nutrition) and choose behaviors subject to time and budget constraints. Let *N* denote child nutritional status (e.g., HAZ), *C* denote consumption/food inputs, *W* denote child characteristics, *H* denote household characteristics, *Z* denote community factors, and *ε* denote an error term:

(1) $$N_{i}=n\left(C_{i},W_{i}H_{i},Z_{i},\varepsilon_{i}\right)$$

The reduced form we estimate writes HAZ as a function of exogenous or predetermined covariates (*W*, *H*, and *Z*):

(2) $${\mathrm{HAZ}}_{i}=f\left(W_{i},H_{i},Z_{i},\varepsilon_{i}\right)$$

#### 2.4.3 Empirical Specification

Let $g\in \left\{1,2\right\}$ index the two periods (*g* = 1 for 2010; *g* = 2 for 2022). We estimate separate linear models

(3) $$y_{i}=X_{i}^{,}\beta_{g}+\varepsilon_{i}$$

where $y_{i}$ is HAZ, $X_{i}$ includes child, maternal, household, and WASH covariates, and $\beta_{g}$ are period‐specific slopes. Denote sample means by *ȳg* = *X̄g'βg*; the overall change is Δ *≡ ȳ₂ − ȳ*₁.

#### 2.4.4 Decomposition (Threefold)

We decompose Δ into (i) an endowments component (E), (ii) a coefficients component (C), and (iii) an interaction (I). Using period 2 (2022) as the reference for E (matching our Stata estimation), the standard threefold form is:

(4) $$∆=\left({\bar{X}}_{2}-{\bar{X}}_{1}\right)\beta_{2}+{\bar{X}}_{1}\left(\beta_{2}-\beta_{1}\right)+\left({\bar{X}}_{2}-{\bar{X}}_{1}\right)\left(\beta_{2}-\beta_{1}\right)$$

E (composition) C (returns) I (interaction).

Negative values indicate contributions that help explain the observed improvement by 2022 (since Δ < 0 in our data); positive values offset the improvement. For robustness, the twofold parameterizations can also be reported, but our main results emphasize the threefold breakdown (Neumark 1988; Oaxaca and Ransom 1994).

#### 2.4.5 Estimation and Inference

We implement weighted least squares with probability weights (popweight) and report robust (Huber–White) standard errors. Decompositions are computed with the Stata module oaxaca (Jann 2008, 2019) using by(year), detail, and vce(robust), which produces the E, C, and I components and variable‐level contributions. The pooled decomposition uses the full sample; regional decompositions are estimated within region subsamples. Because the analysis is observational and relies on linear functional form, results are interpreted as associative components rather than causal effects.

#### 2.4.6 Validation and Robustness

The validity of the findings is supported by several complementary considerations. The analysis uses nationally representative Living Conditions Monitoring Survey data for 2010 and 2022, collected using standardized sampling and survey instruments, with probability weights applied to reflect population‐level patterns. Model specification is grounded in the UNICEF conceptual framework and established empirical evidence on the determinants of child stunting, ensuring theoretical and policy relevance. Robustness is assessed by estimating alternative Blinder–Oaxaca reference structures (Black–White and Gray models) to address the index‐number problem, with consistent results across specifications. In addition, the decomposition results are internally consistent with observed descriptive trends and align with existing evidence on the roles of dietary diversity, caregiving, and environmental health in shaping child growth outcomes, supporting the credibility of the conclusions.

## 3 Results

### 3.1 Descriptive Statistics

**FIGURE 3:**
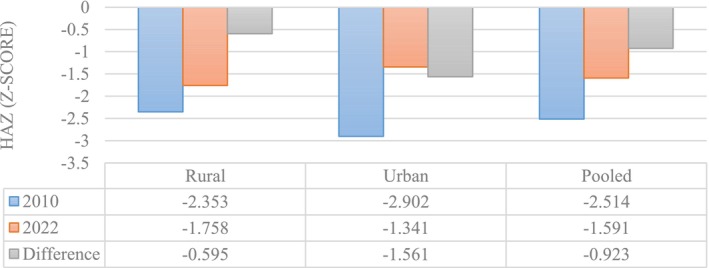
Changes in HAZ between 2010 and 2022 disaggregated by region.

Figure 3 compares mean HAZ scores for the period before the policy change (2010) and the period after the policy change (2022) for the national level, as well as disaggregated by rural and urban areas. The results show that HAZ scores improved nationally (−0.93 SD). Disaggregation by region also shows improvements for both rural and urban areas, though the improvement in urban areas (relative to the baseline) was larger (−1.56 SD) than that for rural areas (−0.60 SD). These differences motivate decompositions by rural and urban to diagnose whether composition (endowments) or returns (coefficients) explain the larger improvement in urban areas.

Table 1 shows the differences in mean values for some key indicators across households and children (0–59 months) between 2010 and 2022 aggregated at national level as well as disaggregated by rural and urban areas. Nationally, the period 2010–2022 shows clear gains likely favorable for height‐for‐age. For example, dietary diversity rose (pooled +0.78 food groups) and WASH coverage expanded sharply (improved water +26 pp; improved sanitation +18.7 pp), with maternal schooling also increasing (+0.79 grades). These factors typically reduce infection exposure and enhance diet quality and care, consistent with the observed improvement in HAZ. At the same time, there are offsetting pressures: the share of expenditure on food increased (+9 pp), suggesting tighter household budgets for nonfood inputs; crowding worsened (+0.08), and the child‐to‐adult ratio climbed (+0.13), all of which can dilute resources and raise infection risk.

**TABLE 1:** Differences in mean values of underlying determinants of HAZ before and after policy change for rural and urban areas.

Category	Variable	Pooled			Rural			Urban		
		2010	2022	Difference	2010	2022	Difference	2010	2022	Difference
Household food security	Food share (mean)	61.21	70.23	9.02***	66.03	74.90	8.87***	49.95	62.52	12.57***
	Dietary diversity score (mean)	4.76	5.53	0.78***	4.39	5.20	0.81***	5.62	6.09	0.47***
Care for mothers and children	Mother's highest grade (mean)	6.94	7.74	0.79***	6.22	6.70	0.47***	8.44	9.32	0.88***
	Mother's age in years (mean)	33.19	32.45	0.74**	33.22	31.54	1.68***	33.12	33.92	0.79
	Share of children to adults (mean)	0.37	0.50	0.13***	0.38	0.52	0.13***	0.33	0.46	0.13***
	Age of child in months (mean)	25.20	27.81	2.61***	24.78	27.26	2.48***	26.17	28.70	2.52**
Healthy environment and health services	Household headed by a female (%)	13.81	22.60	8.79***	12.97	23.29	10.32***	15.76	21.44	5.68***
	Improved source of drinking water (%)	59.95	86.07	26.12***	51.5	85.9	34.4***	49.62	73.68	24.06***
	Improved sanitation (%)	17.78	36.52	18.74***	7.13	21.40	14.27***	42.13	61.72	19.59***
	Household is extremely poor (%)	48.00	45.75	−2.25*	60.73	61.76	1.03	18.25	19.23	0.98
	Crowding index (mean)	0.46	0.53	0.08***	0.43	0.49	0.06***	0.52	0.60	0.08***

*Note:* *, **, and *** denote statistical significance at the 10%, 5%, and 1% levels, respectively.

Rural–urban disaggregation shows that urban areas maintained advantages in sanitation and maternal schooling (and widened both gaps), while rural areas caught up substantially in improved water and diet quality but remain far behind on sanitation, implying persistent enteric disease exposure that can blunt linear growth. Maternal age patterns diverged (younger in rural, slightly older in urban), with potential implications for ANC use and caregiving capacity. Netting these signals, the environment for child growth improved overall, driven by better diets and service access, but residual WASH disparities, rising crowding, and higher food budget shares are likely restraining further gains in HAZ, especially in rural settings where sanitation coverage remains low.

### 3.2 Decomposition Analysis Results

To quantify the contribution of selected predictors in explaining the rural–urban gap in the mean HAZ for the periods before and after the policy change (i.e., 2010 and 2022), we use the Blinder–Oaxaca decomposition analysis. First, we check for evidence of differences in the effects of the determinants using separate regression models for the nationally representative data (pooled) as well as disaggregated by rural and urban areas on the determinants, interacting with the dummy variable separating the periods 2010 and 2022. The joint test on the interaction effects was significant indicating that a Blinder–Oaxaca decomposition is applicable in this context. The estimated models for the rural and urban areas are used to decompose the HAZ gap into explained and unexplained components (Table 2).

**TABLE 2:** Oaxaca decomposition analysis showing mean differences in HAZ under‐five children between 2010 and 2022.

Parameter	Pooled		2010		2022	
	*z*‐score	Std. error/% share	*z*‐score	Std. error/% share	*z*‐score	Std. error/% share
Pooled—Differential						
Mean prediction (2010)	−2.51***	0.07	−2.35***	0.08	−2.90***	0.11
Mean prediction (2022)	−1.59***	0.08	−1.76***	0.11	−1.34***	0.12
Raw difference (2010–2022)	−0.92***	0.11	−0.59***	0.14	−1.56***	0.16
Pooled—Decomposition (threefold displayed; shares vs. gap)						
Explained (endowments)	−0.33**	35.59%	−0.00	0.69%	−0.66***	42.22%
Unexplained (coefficients + interaction)	−0.59	64.41%	−0.59	99.31%	−0.90	57.78%
Coefficients	−0.66***		−0.42**		−0.59**	
Interaction	0.07		−0.17		−0.31	

*Note:* Shares are relative to the raw gap (2010–2022). Significance stars apply to the individual components. *, **, and *** denote statistical significance at the 10%, 5%, and 1% levels, respectively.

The upper panels of Table 2 (Differential) show that mean HAZ improved between 2010 and 2022 in the pooled sample and in both regions. For example, pooled mean HAZ increased from −2.514 (SE 0.070) to −1.591 (SE 0.085), a raw difference of −0.923 (SE 0.110; *p* < 0.001). Rural areas improved by −0.595 (SE 0.143; *p* < 0.001), while urban areas improved by −1.561 (SE 0.170; *p* < 0.001). The lower panels of Table 2 decompose these gaps. Nationally, endowments (E) account for −0.329 of the −0.923 gap (≈36%), and the unexplained component (coefficients + interaction) accounts for −0.595 (≈64%). In rural areas, the gap is primarily driven by the unexplained part (≈99%) with negligible endowments (≈1%). In urban areas, both endowments (≈42%) and unexplained components (≈58%) contribute to the observed improvement.

The detailed decomposition results (Table 3) provide information on the relative contribution of individual covariates to the child nutrition outcomes gap before the policy change (2010). Negative entries indicate contributions that help explain the improvement by 2022 (given the gap definition), while positive entries offset it. The explained and unexplained effects have been grouped according to the respective categories of household food security status, care for mothers and children, and a healthy environment and health services. Among endowments, the largest single contribution comes from HDDS (31.4% of total E), followed by the share of children in the household (58.9% of total E). Improved water offsets the endowment effect (−31.0% of total E). On the returns margin, HDDS again dominates (its coefficient accounts for the bulk of total C), whereas improved water and rooms per person have positive coefficients, offsetting some of the improvement. The constant term is negative, indicating that part of the returns‐based change is captured by the intercept.

**TABLE 3:** Blinder–Oaxaca decomposition: contribution to overall gap between 2010 and 2022 (pooled).

Category	Variable	Endowment effect		Coefficient effect	
		*z*	% of total E	*z*	% of total C
Household food security	Food expenditure share (%)	−0.0563	17.14	−0.2523	37.88
	HDDS	−0.1664***	50.66	−1.7413	261.50
	Household is extremely poor (%)	−0.0175	5.33	0.0098	−1.47
	Category contribution to total	−0.2402	73.13	−1.9838	297.91
Care for mothers and children	Mother's education (index)	−0.0086	2.63	0.0591	−8.87
	Mother's age (years)	0.0064	−1.95	0.1951	−29.30
	Share of children in HH	−0.1935**	58.90	−0.0156	2.34
	Child age (months)	0.0568	−17.28	0.4329**	−65.00
	Household headed by a female	−0.0067	2.03	−0.0082	1.23
	Category contribution to total	−0.1456	44.33	0.6633	−99.61
Healthy environment and health services	Improved source of drinking water	0.1018*	−31.00	0.6423	−96.46
	Improved sanitation	−0.0486	14.79	−0.1376	20.66
	Rooms per person	0.0041	−1.26	0.5239**	−78.68
	Category contribution to total	0.0574	−17.46	1.0287	−154.48
	Constant (coefficients)			−0.3741	
Total		−0.3285	100.00	−0.6659	100.00

*Note:* *, **, and *** denote statistical significance at the 10%, 5%, and 1% levels, respectively.

Table 4 presents the detailed decomposition results for the rural areas. Relative to the pooled results, the rural gap in mean HAZ is smaller (−0.595, *z* = −4.14, *p < 0.001*) and is driven predominantly by coefficients (C = −0.420; ≈70.6% of the gap), with endowments essentially nil (E = −0.004; ≈0.7%) and the remainder from interactions (I = −0.171; ≈28.7%).^1^ Consistent with the national pattern, household dietary diversity (HDDS) is the leading contributor: E = −0.213 (*p* = 0.007) and C = −1.446 (*p* = 0.020), both reducing the gap (i.e., supporting the improvement), while its interaction is positive (I = + 0.240, *p* = 0.021), partially offsetting the net HDDS effect. Improved water shows mixed signals: the coefficient is positive (C = +1.223, *p* = 0.017), offsetting the improvement, but the interaction is negative (I = −0.587, *p* = 0.017), reinforcing it. Child age contributes small but statistically significant offsets via both endowments and coefficients (E = +0.078, *p* = 0.018; C = +0.580, *p* = 0.044). Other covariates are either small in magnitude or imprecisely estimated. Overall, in rural areas the narrowing of the HAZ gap is explained mainly by changes in returns to observed characteristics—especially a stronger HDDS–HAZ gradient—rather than by compositional shifts in endowments.

**TABLE 4:** Blinder–Oaxaca decomposition: contribution to overall gap between 2010 and 2022 (rural areas).

Category	Variable	Endowment effect		Coefficient effect	
		*z*	% of total E	*z*	% of total C
Household food security	Food expenditure share (%)	0.0221	−537.13	−0.8989	214.18
	HDDS	−0.2126***	5155.78	−1.4456**	344.46
	Poor (= 1)	−0.0023	56.83	−0.0617	14.70
	Category contribution to total	−0.1928	4675.48	−2.4061	573.33
Care for mothers and children	Mother's education (index)	0.0015	−35.65	0.2931	−69.84
	Mother's age (years)	0.0218	−529.50	−0.0437	10.40
	Share of children in HH	−0.1937	4698.49	−0.2936	69.97
	Child age (months)	0.0779**	−1889.64	0.5803**	−138.27
	Female (= 1)	0.0005	−11.47	−0.0147	3.51
	Category contribution to total	−0.0920	2232.23	0.5213	−124.22
Healthy environment and health services	Improved drinking water (= 1)	0.2781	−6745.56	1.2234**	−291.52
	Improved sanitation (= 1)	0.0101	−246.16	−0.1045	24.91
	Rooms per person	−0.0076	184.01	0.0834	−19.86
	Category contribution to total	0.2807	−6807.71	1.2022	−286.47
	Constant (coefficients)			0.2629	
Total		−0.0041	100.00	−0.4197	100.00

*Note:* *, **, and *** denote statistical significance at the 10%, 5%, and 1% levels, respectively.

Table 5 presents the detailed decomposition results for the urban areas. The mean HAZ gap is large (−1.561; *z* = −9.19; *p* < 0.001), with both endowments and coefficients contributing significantly: E = −0.659 (≈42%) and C = −0.586 (≈38%), while the interaction is I = −0.315 (≈20%; n.s.). The standout determinant is housing space: rooms per person has a large positive coefficient effect (C = +1.198, *p* = 0.008), which offsets part of the overall improvement (i.e., the return to housing space moved in a direction that raises the gap), but this is partly counteracted by a negative interaction (I = −0.176, *p* = 0.013), implying that concurrent changes in levels and returns modestly reinforced the improvement. On the composition side, household structure and spending matter: a higher child share (E = −0.253, *p* = 0.066) and a lower food expenditure share (E = −0.214, *p* = 0.088) both narrow the gap (marginal significance), suggesting that urban areas' gains are linked to changes in dependency ratios and budget allocation. Other endowment and coefficient terms are smaller or imprecisely estimated. Overall, in urban areas, the narrowing of the HAZ gap reflects a joint role of changing characteristics and changing returns, with the returns to housing space emerging as the principal offsetting force.

**TABLE 5:** Blinder–Oaxaca decomposition: contribution to overall gap between 2010 and 2022 (urban areas).

Category	Variable	Endowment effect		Coefficient effect	
		*z*	% of total E	*z*	% of total C
Household food security	Food expenditure share (%)	−0.2138*	32.44	−0.0054	0.92
	HDDS	−0.0312	4.74	−0.3121	53.21
	Poor (= 1)	0.0001	−0.01	−0.0221	3.77
	Category contribution to total	−0.2449	37.16	−0.3395	57.89
Care for mothers and children	Mother's education (index)	−0.0269	4.08	−0.1702	29.02
	Mother's age (years)	−0.0057	0.86	0.7380	−125.84
	Share of children in HH	−0.2530*	38.38	0.1794	−30.59
	Child age (months)	0.0153	−2.32	0.3234	−55.14
	Female (= 1)	−0.0168	2.54	0.0305	−5.20
	Category contribution to total	−0.2870	43.54	1.1011	−187.75
Healthy environment and health services	Improved drinking water (= 1)	−0.0266	4.03	−0.2896	49.38
	Improved sanitation (= 1)	−0.1122	17.02	0.1241	−21.16
	Rooms per person	0.0116	−1.76	1.1979***	−204.25
	Category contribution to total	−0.1272	19.30	1.0324	−176.03
	Constant (coefficients)			−2.3805	
Total		−0.6591	100.00	−0.5865	100.00

*Note:* *, **, and *** denote statistical significance at the 10%, 5%, and 1% levels, respectively.

## 4 Discussion and Implications for Policy

In this study, we systematically investigate the underlying factors that accounted for the changes in child stunting in Zambia and how these differed across rural and urban areas before and after the changes in the food and nutrition policy strategy in 2011 and 2017. Nationally, mean height‐for‐age *z*‐scores (HAZ) improved between 2010 and 2022 (pooled Δ ≈ −0.92 SD), alongside broad gains in improved drinking water (+26 pp), improved sanitation (+18.7 pp), dietary diversity (+0.78 food groups), and maternal schooling (+0.79 grades), but with rising crowding (+0.08), a higher child‐to‐adult ratio (+0.13), and larger food budget shares (+9 pp). This pattern aligns with Zambia's shift in food and nutrition policy—beginning in 2011—to a multisectoral strategy focused on the first 1000 days after conception, stronger community delivery, and explicit WASH–nutrition linkages under the National Food and Nutrition Strategic Plans (NFNSP 2011–2015; NFNSP 2017–2021) and the Most Critical Days Programme (MCDP) (NFNC 2011, 2017).

The magnitude and pattern of improvements observed in this study are broadly consistent with, but also extend, existing evidence on child stunting in Zambia and comparable settings. The national improvement in mean height‐for‐age *z*‐scores between 2010 and 2022 aligns with documented declines in stunting prevalence reported in recent demographic surveys, which show persistent but narrowing gaps between rural and urban areas (CSO et al. 2014; ZamStats et al. 2023; ZamStats et al. 2024). Like earlier studies, maternal education, dietary quality, and WASH‐related factors emerge as important correlates of child growth (Black et al. 2013). However, by decomposing changes over time, the present study shows that recent gains—particularly in rural areas—are driven more by stronger returns to dietary diversity than by improvements in underlying endowments, a distinction not captured in cross‐sectional analyses. In urban areas, the joint role of compositional change and changing associations is consistent with findings from multi‐country studies that highlight the growing importance of environmental and housing‐related constraints in dense urban settings (Headey et al. 2017). Together, these comparisons suggest that while Zambia's nutrition trends follow regional patterns, the pathways through which improvements occur differ by context, underscoring the added value of the decomposition approach used in this study.

Disaggregating by region reveals distinct mechanisms of change. In rural areas, the improvement is returns‐driven (≈71% of the gap), with negligible composition; the household dietary diversity score (HDDS) shows a dominant negative (beneficial) coefficient contribution, implying that diet quality paid off more for linear growth by 2022—consistent with intensified first‐1000‐days counseling, infant and young child feeding (IYCF), and community outreach under MCDP. Persistently low rural sanitation, however, likely constrained further gains (NFNC 2011, 2017). In urban areas, the much larger HAZ improvement reflects a joint role of endowments (~42%) and returns (~38%). On the composition side, shifts in household structure (child share) and budget allocation (food share) contributed to narrowing the gap, resonating with NFNSP calls to align social protection, livelihoods, and food systems to support affordable, diverse diets. On the returns side, the housing‐space coefficient is positive (offsetting), suggesting that improvements in rooms per person did not fully translate into growth—plausibly due to exposure and environmental‐health constraints in denser settings—an implementation challenge flagged in the 2017–2021 NFNSP and MCDP II (NFNC 2017).

Overall, the two NFNSPs and the MCDP phases provided the architecture and program content (1000‐day focus; WASH–nutrition integration; district convergence; Coordination) needed to move multiple determinants together. The data are directionally consistent with these policies: diet quality and service coverage improved, and returns to some inputs (notably diets) strengthened; yet sanitation gaps in rural areas, crowding/quality constraints in urban areas, and rising food budget pressure likely tempered the full HAZ response—underscoring the need for coordinated, quality‐assured delivery and monitoring (NFNC 2011, 2017).

The findings of this study generate several policy‐relevant insights for nutrition and health programming in Zambia, particularly in relation to persistent rural–urban disparities in child stunting.

First, the results reinforce the need to move away from uniform national nutrition programmes towards more site‐specific, context‐sensitive interventions. Although child height‐for‐age improved nationally between 2010 and 2022, the decomposition results show that the mechanisms underlying these improvements differ substantially between rural and urban areas. In rural areas, gains are driven almost entirely by stronger returns to existing household characteristics—most notably dietary diversity—rather than by improvements in endowments. This implies that nutrition counseling and behavior change interventions have become more effective, but that structural constraints remain. Future rural nutrition policies should therefore combine continued emphasis on diet quality with targeted investments in underlying conditions that enable dietary improvements to translate into sustained child growth.

Second, the findings highlight sanitation and environmental health as binding constraints on rural nutrition outcomes. While access to improved drinking water expanded markedly over the study period, rural sanitation coverage remains low. The results suggest that continued exposure to poor sanitation and hygiene likely dampens the nutritional returns to improved diets by increasing infection‐related nutrient losses. From a nutrition policy perspective, this points to the need for stronger integration of nutrition‐specific interventions with sanitation and hygiene behavior change, ensuring that improvements in dietary intake are biologically effective in promoting child linear growth.

Third, the urban results point to the importance of environmental and housing‐related factors in shaping child nutrition outcomes. Urban areas experienced larger improvements in child height‐for‐age, driven by both improved household endowments and changes in returns. However, the positive coefficient on housing space indicates that improvements in living space have not consistently translated into proportional gains in child growth. This suggests that urban environmental risks—such as inadequate sanitation, water quality concerns, and high population density in informal settlements—may offset the potential nutritional benefits of improved housing. Urban nutrition policy should therefore be complemented by interventions that strengthen environmental health and food safety at the community level, particularly in high‐density urban settings.

Fourth, rising food expenditure shares across both rural and urban households indicate increasing pressure on household food budgets, with implications for diet quality and nutritional adequacy. Higher food budget shares may limit households' ability to maintain diverse and nutrient‐rich diets, especially during periods of economic stress. These findings support policies that protect and deepen diet quality through nutrition‐sensitive food system actions—such as promoting diversified food production, improving market access for nutrient‐dense foods, and linking nutrition programming with social protection mechanisms that help households absorb food price shocks.

Overall, the results suggest that Zambia's multisectoral nutrition strategy has contributed to improvements in child nutrition, but that further reductions in stunting will require context‐specific policy responses. In rural areas, priority should be given to improving sanitation and hygiene to enhance the effectiveness of dietary improvements, while in urban areas, nutrition gains will depend increasingly on addressing environmental health and food safety challenges. Tailoring nutrition and health policies to these distinct rural and urban pathways is essential for accelerating progress and reducing spatial inequalities in child stunting.

## 5 Conclusion

This study assessed changes in child height‐for‐age in Zambia between 2010 and 2022 using nationally representative survey data and a Blinder–Oaxaca decomposition framework. Child nutritional status improved over the period, but through different pathways across rural and urban areas. In rural settings, improvements were driven mainly by stronger returns to dietary diversity, with limited progress in underlying conditions, while in urban areas gains reflected both improved household characteristics and changing associations, partly offset by environmental and housing‐related constraints. By decomposing changes in child growth rather than focusing on static correlates, the study provides novel evidence on how nutrition pathways have evolved over time and how these pathways differ spatially. The findings underscore the importance of context‐specific nutrition and health strategies that address both diet quality and the environmental conditions that shape its effectiveness. The results are directly relevant to the Sustainable Development Goals. Improvements in child growth support progress towards SDG 2 (Zero Hunger) and SDG 3 (Good Health and Well‐being), while persistent rural–urban differences highlight the importance of SDG 10 (Reduced Inequalities). Overall, the evidence supports integrated and geographically targeted nutrition and health policies as a means of accelerating reductions in child stunting and advancing global development objectives.

## 6 Limitations of the Study

This study has several limitations that should be considered when interpreting the findings.

First, the analysis relies on repeated cross‐sectional data rather than longitudinal panel data. Although the Living Conditions Monitoring Surveys (LCMS) provide nationally representative information at two points in time, the same households and children are not followed over time. As a result, the observed changes in child height‐for‐age z‐scores and their decomposed components reflect population‐level associations rather than individual growth trajectories. The Blinder–Oaxaca decomposition therefore captures how changes in characteristics and their associations coincide with improved nutrition outcomes, but it does not permit causal inference about the effects of specific policies or interventions.

Second, the study is subject to potential measurement and reporting biases inherent in household survey data. Key variables such as food consumption, dietary diversity, and household expenditure are self‐reported and may be affected by recall error or social desirability bias. While the LCMS uses standardized survey instruments and trained enumerators to minimize such biases, some degree of measurement error is unavoidable and may attenuate estimated relationships.

Third, data availability constraints limit the set of nutrition‐relevant variables that can be included in the analysis. Although the LCMS offers richer information on household food security and living conditions than many alternative surveys, it lacks detailed data on some important determinants of child nutrition, such as maternal nutritional status, infant feeding practices at the individual child level, morbidity episodes, and micronutrient intake. The omission of these factors may result in unmeasured confounding, particularly if such variables are correlated with both observed determinants and child growth outcomes.

Fourth, the interpretation of the decomposition results is sensitive to model specification and functional form assumptions. The Blinder–Oaxaca framework assumes linear relationships between covariates and nutritional outcomes and decomposes differences based on estimated coefficients. While this approach is well established in the nutrition and development literature, it may not fully capture nonlinearities or complex interactions among determinants, especially across heterogeneous rural and urban contexts.

Finally, although the analysis aligns changes in nutritional outcomes with the timing of Zambia's multisectoral nutrition strategies, the study does not directly measure programme exposure or implementation intensity. As such, improvements in child nutrition cannot be attributed exclusively to specific policy instruments, but rather reflect the combined influence of broader economic, social, and policy changes over the study period. Despite these limitations, the study provides robust and policy‐relevant evidence on how key nutrition‐related determinants and their associations with child growth have evolved over time, offering valuable insights for the design of context‐sensitive nutrition and health policies in Zambia.

## Author Contributions

**Thomson Kalinda:** supervision, funding acquisition, conceptualization, methodology. **Richard Bwalya:** conceptualization, methodology, data curation, funding acquisition, investigation.

## Funding

This work was supported by the African Economic Research Consortium (AERC) under the Collaborative project on “Agricultural and Food Policy Analysis for Nutrition Outcomes in Africa” [Grant Number RC18519].

## Conflicts of Interest

The authors declare no conflicts of interest.

## Data Availability

The data supporting the findings of this study are available from the Zambia Statistics Agency (ZamStats) subject to approval. An anonymized dataset and analysis code are available at: [data files and analysis code].

## Endnotes

^1^
Differences are defined as 2010–2022 (negative values indicate improvement). Percentage shares are component/total (E/Δ, C/Δ, I/Δ).
